# Minimally Invasive Mitral Valve Surgery: A Systematic Review

**DOI:** 10.1155/2013/179569

**Published:** 2013-03-27

**Authors:** Fabiana Lucà, Leen van Garsse, Carmelo Massimiliano Rao, Orlando Parise, Mark La Meir, Calogero Puntrello, Gaspare Rubino, Rocco Carella, Roberto Lorusso, Gian Franco Gensini, Jos G. Maessen, Sandro Gelsomino

**Affiliations:** ^1^Cardiothoracic and Cardiology Department, Maastricht University, The Netherlands; ^2^Heart and Vessels Department, Careggi Hospital, Florence, Italy; ^3^Cardiology Department, Paolo Borsellino Hospital, Marsala, Italy

## Abstract

In the recent years minimally invasive mitral valve surgery (MIMVS) has become a well-established and increasingly used option for managing patients with a mitral valve pathology. Nonetheless, whether the purported benefits of MIMVS translate into clinically important outcomes remains controversial. Therefore, in this paper we provide an overview of MIMVS and discuss results, morbidity, mortality, and quality of life following mitral minimally invasive procedures. MIMVS has been proven to be a feasible alternative to the conventional full sternotomy approach with low perioperative morbidity and short-term mortality. Reported benefits of MIMVS include also decreased postoperative pain, improved postoperative respiratory function, reduced surgical trauma, and greater patient satisfaction. Finally, compared to standard surgery, MIMVS demonstrated comparable efficacy across a range of long-term efficacy measures such as freedom from reoperation and long-term survival.

## 1. Introduction

Minimally invasive mitral valve surgery (MIMVS) has been proven as a feasible alternative to conventional full sternotomy approach with low perioperative morbidity and short-term mortality [1, 2]. As a result, MIMVS is being employed increasingly as routine approach in many centers worldwide with excellent short-term and long-term results [3, 4]. 

During the past years, several studies on outcomes of MIMVS have been published in the literature [5–7]. Furthermore, since the first description of MIMVS by Cohn et al. [8] and Navia and Cosgrove [9] in the mid 1990s, various minimally invasive approaches have been reported including the parasternal, hemisternotomy, minithoracotomy, and totally endoscopic approaches [10–12]. However, despite the differences in surgical approaches, the shared goal of all these MIMVS procedures is to avoid median sternotomy-related complications such as infection, mediastinitis, and nerve injuries [8, 13–19] and, at the same time, to provide a safe and effective option for mitral valve surgery with the clinical benefits associated with a minimal access approach.

Nonetheless, whether the supposed benefits of MIMVS translate into clinical favorable outcomes still remains controversial, and there are conflicting opinions about whether minimally invasive surgery is ready for routine uptake in place of conventional open mitral valve surgery. 

In this paper we provide an overview of MIMVS and discuss results, morbidity, mortality, and quality of life following mitral minimally invasive procedures.

## 2. Review Criteria

Papers selected for this review were identified on PUBMED using the search terms “minimally invasive mitral valve surgery.”

All articles were reviewed and references were selected on the basis of historical contribution, number of patients, and new contributions to the field.

## 3. Surgical Procedure

**Figure 1:**
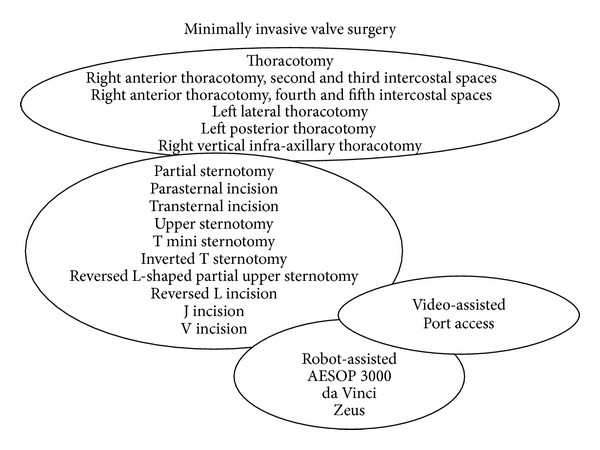
Minimally invasivemitral valve surgery: techniques overview.

MIMVS refers to a constellation of surgical techniques/technologies (Figure 1) that minimize surgical trauma through smaller incisions compared with a conventional sternotomy. The most common minimally invasive approach to the mitral valve includes a right minithoracotomy [8], a robotically assisted right thoracic approach [20], and a partial sternotomy [21]. 

In 1923 Elliot Carr Cutler, in conjunction with his cardiology colleague, Samuel Levine, performed a closed transventricular mitral commissurotomy on a 12-year-old patient with rheumatic mitral stenosis at the Peter Bent Brigham Hospital. 

The patient survived surgery but died of pneumonia 4 years postoperatively. In the following years, Cutler performed seven more operations using his new cardiovalvulotome. Unfortunately, this concept did not promote long-term success and a moratorium for these operations was called in 1929. However, this pioneering effort in 1923 was the first successful operation to treat valvular heart disease by a surgical technique [22].

A transseptal approach to the mitral valve was described by Dubost and colleagues [23] using a biatrial incision and transecting the septum whereas Guiraudon and associates [24] described an approach via the right atrium. 

By the mid 1990s, the success of laparoscopic operations in general surgery renewed an interest in minimally invasive approaches for cardiac surgery. During April and May 1996, minimally invasive mitral valve operations were performed on 25 patients by Navia and Cosgrove [8, 9]. All patients underwent mitral valve repair performed through a small right parasternal incision. Although the surgical field is smaller than a median sternotomy, the mitral valve is positioned in the center of the incision, and, if the atrium is small, extension of the incision over the dome of the left atrium provides a substantial improvement of exposure. There were no hospital deaths, reoperations for bleeding, embolic complications, wound infections, or valve repair failures. No sinus node dysfunction or atrioventricular dissociation resulted [9]. 

From 1996 to 1997, Cohn et al. [8] presented 84 minimally invasive cases (41 aortic, 43 mitral) using a right parasternal incision and excising the third and fourth costal cartilage. For mitral valve replacement or repair, all incisions were performed through a right parasternal incision, excising the third and fourth costal cartilage. The right atrium was exposed and opened after caval tapes were put down, isolating the right atrium. The aortic cross-clamp was applied before incising the right atrium. A transseptal incision then was made into the left atrium. Once the atrial septum was incised, the mitral valve was repaired or replaced by standard techniques [25, 26].

The operative mortality for mitral valve surgery was 0 (0%) of 43. There had been no perivalvular leaks in any of the valves implanted, and there has been excellent visualization of the mitral valves as to perform complicated repairs, including leaflet resection, chondroplasty, and commissuroplasty documented by intraoperative and postoperative transesophageal echo [8]. Smaller incisions lateral to the sternum have been introduced, with or without resection of the third or fourth costal cartilage. However, their disadvantages included femoral CPB cannulation, ligation of the right internal thoracic artery, occasional chest wall instability, and difficult conversion to full sternotomy [4].

In 1996, Carpentier et al. [27] performed the first video-assisted mitral valve repair through a minithoracotomy using ventricular fibrillation.

From 1996 to 1998 the Leipzig group [28] studied one hundred and twenty-nine patients with nonischemic mitral valve disease undergoing 3D video assisted mitral valve surgery via a 4 cm right lateral minithoracotomy using femorofemoral bypass and endoaortic clamping. After the initial series (group I, *n* = 62), a voice controlled robotic arm (AESOP 3000, Automated Endoscope System for Optimal Positioning; Computer Motion, Santa Barbara, CA) was employed to guide the video scope in the last series (group II, *n* = 67). Finally, intraoperative transesophageal echocardiography was introduced for real-time monitoring of cardiac distention, deairing, and cannula placement [29].

Felger et al. [30] evaluated a series of video-assisted minimally invasive mitral operations, showing safe progression toward totally endoscopic techniques. Consecutive patients with isolated mitral valve disease underwent either manually directed (*n* = 55) or voice-activated robotically directed (*n* = 72) video-assisted mitral operations. The consecutive series was evaluated in five cohorts comparing serial cross-clamp and perfusion times. Cold blood cardioplegia, a transthoracic aortic clamp, a 5 mm endoscope, and a 5 cm minithoracotomy were used. This video-assisted minimally invasive mitral operation cohort was compared with a previous sternotomy-based mitral operation cohort (*n* = 100). Repairs were performed in 61.8% manually directed (MD, *n* = 34), 75.0% robotically directed (RD, *n* = 54), and 54% sternotomy-based (*N* = 54) mitral operations. The robotically directed technique showed a significant decrease in blood loss, ventilator time, and hospitalization compared with the sternotomy-based technique. Manually directed mitral operations compared with robotically directed mitral operations had decreased arrest times (128.0 ± 4.5 minutes compared with 90.0 ± 4.6 minutes; *P* < 0.001) and decreased perfusion times (173.0 ± 5.7 minutes compared with 144.0 ± 4.6 minutes; *P* < 0.001). In the minimally invasive mitral operation cohort, complications included reexploration for bleeding (2.4%; *n* = 3) and one stroke (0.8%), whereas the 30-day mortality was 2.3% (*n* = 3). Operative times were significantly less with RD operations versus MD operations (*P* < 0.002) Table 1.

The next evolutionary bound in endoscopic mitral surgery was the development of three-dimensional (3D) vision and computer-assisted telemanipulation that could transpose surgical movements from outside the chest wall todeep within cardiac chambers; in that same year, Carpentier et al. [47] performed the first completely robotic MVR using the Da Vinci Surgical System (Intuitive Surgical,Inc., Sunnyvale, California, USA). Soon after, the East Carolina University group performed the first mitral valve replacement through a minithoracotomy, using video direction [8, 20].

Another promising technique is the Port access for MIMVS [31, 48–50].

Stevens and colleagues at Stanford University introduced in Europe in March 1996 a surgical method for performing Port-access bypass grafting [51].

In 1998, Mohr reported the Leipzig University experience using the Port access technology, which was based on endoaortic balloon occlusion (EABO). The study recruited 51 consecutive patients with nonischemic mitral valve disease who undergone mitral repairment (*n* = 28) or replacement (*n* = 23) by means of a minimally invasive approach through a right lateral minithoracotomy and under videoscopic guidance. Acute retrograde aortic dissection occurred in two patients [50]. Both events were most likely caused by intimal dissection at the level of the iliac artery induced by the guide wire. Retrograde flow led to complete retrograde aortic dissection.

The Port access technology has some complicated aspects such as the introduction and the placement of the endoaortic balloon catheter and its intraoperative monitoring. Transesophageal echocardiography and fluoroscopy are used to verify proper positioning of the coronary sinus and pulmonary artery vent catheters and the venous drainage cannula and endoaortic balloon [52, 53]. During CPB, verification of proper positioning of the endoaortic balloon is vital because proximal migration can damage the aortic valve and distal migration can decrease cerebral perfusion by occluding the brachiocephalic artery [52]. Because distal migration may compromise cerebral blood flow, it is imperative to monitor endoaortic balloon position continuously. Multiple monitoring techniques are used to confirm proper positioning of the endoaortic balloon in the ascending aorta. Transesophageal echocardiography is useful in visualizing the ascending aorta and endoaortic balloon location [54], but it may become difficult to visualize the balloon position when the heart is fully arrested during CPB. 

The implementation of continuous transcranial Doppler flow measurements of the middle cerebral arteries added an important safety measure, as right radial artery pressure measurements alone are not sensitive enough to immediately detect impairment of cerebral perfusion caused by balloon migration to the aortic arch [11]. 

However, the Port access technique still continues to be associated with significant risks such as peripheral CPB cannulation and a high rate of retrograde aortic dissection balloon catheter to occlude the aorta and provide cardioplegia.

An 8 cm anterolateral thoracotomy via the third intercostals space, direct aortic clamping, and cannulation has been described by Angouras and Michler [55].

Telemanipulators, robotics that allow a hand-like mechanism to be controlled by a human operator, were first used by Mohr et al. [28] and Falk et al. [11].

Chitwood et al. [56, 57] and Kypson et al. [58, 59] showed that this technique could be safely and effectively used. Recently, another group reported the results of 25 patients receiving successful telemanipulator-supported MIMVS [60]; however, long-term results are not available. Other centers had similar positive experiences using the telemanipulator-supported techniques in the late 1990s [61, 62]. However, they later abandoned this technique, given the lack of difference compared with their standard approaches. In 2009, Wang et al. [63] presented a new approach for MV replacement through a right vertical infra-axillary thoracotomy with excellent results (0.5% mortality).

## 4. Mortality

After reviewing all comparative miniVS studies evaluating mortality, no study showed a significant difference between minimally invasive and conventional approaches [32, 34, 38, 39, 42, 43, 46, 64]. 

Mihaljevic et al. compared 474 minimally invasive mitral operations (mostly lower sternotomy and right parasternal) with 337 median sternotomy procedures. The perioperative mortality was 0.2% for the minimally invasive group and this is compared favorably with 0.3% in the sternotomy patients. However, the MIMVS patients were found to be a lower risk group (better ejection fraction, more repairs, less symptomatic), and no attempt was made to adjust for these differences [44]; Furthermore, Grossi et al. matched 100 consecutive patients undergoing minimally invasive aortic and mitral valve surgery over a 2.5-year period (through either a 3rd or 4th interspace incision) to patients having the same valve surgery via a sternotomy [38]. They demonstrated no significant difference in hospital mortality (3.7% versus 3.4%, resp.) between groups, even though mean CPB times was 30 min longer in the minimally invasive group. Six studies met the inclusion criteria for our analysis and revealed no significant mortality difference between groups (1,641 patients, OR 0.46, 95% CI 0.15–1.42, *P* = 0.18) [38, 43, 44, 46].

## 5. Neurological Events

Due to the physical limitations of MIMVS, inadequate de-airing leading theoretically to a higher incidence of neurological complications was a primary concern, making the use of transesophageal echocardiography mandatory. In his early series, Mohr [50] reported an 18% incidence of postoperative confusion; however, continuous Co_2_ insufflation was not used, as in more recent series. One decade later, Seeburger et al. [3] observed postoperative neurological impairment in 41 of 1,339 patients (3.1%) who underwent mini MVS, with 28 (2.1%) minor and 13 (1.0%) major events. Ten studies reported no difference in the incidence of stroke [31, 39, 65, 66], while two showed a decreased incidence following a minimally invasive approach [43, 67]. In a systemic metaanalysis [3], there was no significant difference in neurological events in 6 eligible studies including a total of 1,801 patients.

Schneider et al. used transcranial Doppler to detect cerebral microemboli in 21 MIMVS patients undergoing endoaortic balloon occlusion with continuous Co_2_ chest cavity insufflation. These were compared to 14 patients undergoing conventional mitral surgery [36]. They found no significant difference in the cerebral microembolic rate between either technique.

The Consensus Statement of the International Society of Minimally Invasive Coronary Surgery (ISMICS) 2010, based on a systematic review and meta-analysis of all available randomized and nonrandomized comparative trials of isolated mini versus conventional mitral valve surgery (two randomized trials and 33 nonrandomized studies for a total of 35 studies) [68], associated some adverse clinical outcomes with mini MVS compared with conv-MVS, including stroke, aortic dissection, and groin wound/vasculature complications. The absolute risk increase of stroke for mini MVS versus conv-MVS was 0.9% overall (2.1% versus 1.2%, RR 1.79, 95% CI 1.35–2.38; 13 studies, level B). Subanalysis of two propensity comparison studies also showed significant increase of stroke of 1% with mini MVS compared with conv-MVS (1.9% versus 0.9%, RR 2.02, 95% CI 1.40–2.94; two studies, level B) [69].

These findings are similar to those recently reported by a recent Society of Thoracic Surgeons-Adult Cardiac Surgical Database (STS-ACSD) publication made on 28,143 patients undergoing isolated mitral valve operations that examined the associations between operative strategy and the increased risk of stroke in the less-invasive group [70].

The markedly higher rate of permanent perioperative stroke in the less-invasive group compared with the conventional sternotomy group in unadjusted, adjusted, and propensity analyses was the most significant finding of this study. The adjusted OR for permanent stroke was 1.96 for less-invasive compared with conventional sternotomy operations in the multivariable analysis, and the likelihood of stroke was similarly increased in the propensity analysis. Among the 4,322 LIMV operations, there were 41 excess strokes compared with the propensity-matched group having conventional mitral valve operations. Additional analyses demonstrated a threefold higher risk of stroke for less-invasive operations performed without aortic occlusion (beating- or fibrillating-heart), which comprised 12% of the less invasive group. Femoral cannulation was not an independent predictor of stroke [70].

Grossi et al. [71] using an informal strategy of intraoperative echocardiographic analysis of the aortic arch and the descending aorta in 714 minimally invasive mitral valve procedures had excellent results from this approach avoiding the use of femoral perfusion when there was significant atherosclerotic burden [71]. In this cohort, where 30% of patients were >70 years of age, 15% were reoperations, and 12% were multivalve operations, femoral perfusion was used in nearly 80% of patients, with a 2.9% incidence of stroke. Afterwards they developed an aortic cannulation through a minithoracotomy incision that became the “go-to” approach for the majority of our minimally invasive mitral valve procedures, regardless of age.

The same group [72] reviewed a large minimally invasive valve experience using a robust data collection instrument. The study recruited 3,180 patients undergone to isolated, nonreoperative valve operations: 1,452 (45.7%) aortic valve replacements and 1,728 (54.3%) mitral valve procedures. The surgical approach was with standard sternotomy (*n* = 889; 28%) or by minimally invasive techniques (*n* = 2,291; 72%). Antegrade arterial perfusion was used in 2,646 (83.2%) cases and retrograde perfusion was used in 534 (16.8%) cases. Multivariable analysis revealed that age, atherosclerotic aorta, cerebrovascular disease, emergent procedure, ejection fraction less than 0.30, no use of aortic clamp, and retrograde perfusion were significantly associated with stroke. In patients 50 years old or younger (*n* = 662), retrograde perfusion had no significant impact on the incidence of stroke (1.6% versus 1.1%, *P* = 0.57). In this study, minimally invasive approaches for isolated aortic or mitral valve operations did not increase the perioperative risk of stroke when performed with antegrade perfusion. However, the risk of stroke did increase with the use of retrograde perfusion in older patients. Multivariable risk factors for stroke were retrograde perfusion (odds ratio 4.4; *P* < 0.01) and ejection fraction below 0.30 (odds ratio 2.1; *P* = 0.09). The authors concluded that the incidence of stroke in reoperative mitral operations was associated with perfusion strategies and not with the surgical approach [71]. The overall stroke rate was 2.2%, with increased stroke risk associated with an atherosclerotic aorta, cerebrovascular disease, emergent operation, ejection fraction <30% or retrograde perfusion (*P* < 0.05 for each), but not with incision location (*P* = 0.82). Additionally, the association of retrograde perfusion became insignificant when analyzing patients who were 50 years old or younger [72].

These results mirror those of a previous cohort of patients undergoing reoperative mitral valve procedures, which revealed that retrograde perfusion was the only independent risk factor for stroke (odds ratio 4.4; *P* = 0.001) [73].

Later, Grossi and colleagues presented a focused report on a more homogeneous subset of 1,282 first-time, isolated mitral valve operations performed through a right anterior minithoracotomy over a 12-year period [74]. This homogeneity allowed us greater discriminatory power to analyze the specific patient factors associated with an increased risk of stroke. The only significant risk factor interaction for neurologic complication identified was the use of retrograde perfusion in patients with high-risk comorbidities: peripheral vascular disease, cerebrovascular disease, atherosclerotic aortas, or dialysis dependence.

These data suggest that retrograde perfusion remains a viable option for younger patients without vascular comorbidities. In older patients or those with the risk factors discussed previously, performing a computed tomography angiography of the descending aorta with distal runoff in addition to an intraoperative transoesophageal echocardiographic assessment of the thoracic aorta [74, 75] is currently recommend. Such an approach has been shown to be effective by Murphy et al. [76], who demonstrated a 1.6% stroke rate using retrograde perfusion in similarly screened patients undergoing robotic cardiac procedures. Minimally invasive valve surgery with antegrade perfusion has a low risk of neurological complications and has excellent outcomes. Retrograde perfusion in older patients with significant vascular comorbidities is associated with an increased risk of stroke. The vast majority of patients currently undergo heart valve procedures through a right anterior minithoracotomy with antegrade perfusion via direct ascending aorta cannulation obviating the concerns associated with retrograde perfusion. For those procedures in which the direct access to the ascending aorta is extremely limited, in a recent editorials Yaffee et al. [75] recommend preoperative aortic screening to identify aortic pathology and to avoid retrograde perfusion in patients where high atheroembolic risk exists.

## 6. Bleeding, Transfusion, and Reexploration

A reduction in postoperative hemorrhage and transfusion requirements has been suggested as a potential advantage of minimally invasive valve surgery. This benefit is important given the significant morbidity and mortality associated with transfusions and reexploration for bleeding [77]. Smaller incisions should theoretically reduce postoperative bleeding and transfusion requirements, notably with the significant morbidity/mortality associated with transfusions and bleeding reexploration. Some studies report no difference in transfusion requirements [45]. Four comparative studies reported blood loss volume with three utilizing a minithoracotomy [28, 31, 66] and one selecting a parasternal approach [42]. 

Mohr et al. demonstrated no difference in blood loss or blood product transfusions in 31 videoscopic mitral procedures compared with a conventional sternotomy, despite fewer reexplorations for bleeding [28]. The robotically directed technique showed a significant decrease in blood loss as well as ventilator time and hospitalization compared with the sternotomy-based technique [30]. Felger et al. reported that there was no significant difference either in percentage of patients receiving transfusions or in the amount of packed red blood cells, fresh frozen plasma, or platelets transfused; however, postoperative chest tube drainage was significantly less in minimally invasive patients compared with sternotomy patients (*P* = 0.006). Because extreme values skewed the raw data for ventilator hours, a rank order analysis of variance was performed to provide homogeneity of the data. The ranked ventilator hours revealed a significant difference between conventional and minimally invasive patients (*P* = 0.006), but no difference was found between the RD and MD patients (*P* = 0.984). All three cohorts had similar intensive care unit lengths of stay (*P* = not significant). However, length of stay from operative procedure to discharge was significantly less in the RD and MD cohorts compared with conventional cohorts (*P* = 0.001). In all minimally invasive mitral valve operations the bleeding was controlled through the thoracotomy incision without the need for extension. However, there was no significant difference either in the percentage of patients receiving transfusions or the amount of blood products transfused [30] In addition, in a prospective, randomized trial, Dogan et al. [45] found a significant decrease in postoperative chest tube output in the miniVS group compared with the conventional group. In a consecutive series of 41 patients undergoing either Port access (*n* = 21) or sternotomy (*n* = 20) mitral surgery, Glower et al. demonstrated no significant difference in chest tube drainage or transfusion requirements despite longer CPB times in the former [31]. Grossi et al. [39] found that a right thoracotomy was associated with 51% fewer blood products than a conventional sternotomy.

In robotically assisted MVR, transfusion requirements are even lower (20% to 45% require transfusions) [11, 78]. Furthermore, 4 comparative studies found less blood loss: a minithoracotomy was used in 3 [26, 30, 31] and a parasternal approach was used in 1 [42]. Three of 10 studies found reduced transfusion requirements with a minimally invasive approach compared with conventional surgery [8, 34, 38] whereas the others showed no difference [31, 33, 42, 46, 65, 67, 77].

More convincing evidence came from a subsequent study by the same group that showed 13% fewer total transfusions with 1.8 fewer units of red blood cells using a minithoracotomy compared to a sternotomy [39].

Similar data from Cohn et al. confirm that patients undergoing minimally invasive valve surgery are transfused 1.8 units less compared to a conventional cohort [8]. Two of seven studies [56, 65] demonstrated a reduced need for reoperation for bleeding with a minimally invasive approach [38, 42, 44, 46]. Further, 5 studies showed a significant reduction in reoperations for bleeding with a minimally-invasive approach [32, 38, 42–44, 49, 64]. The recent data from the Leipzig group on postoperative course included reoperation for bleeding in 69 patients (5.1%) [3].

## 7. Atrial Fibrillation

It has been suggested that a less traumatic surgical approach would be a less potent trigger of postoperative AF. Nonetheless, 5 of 6 studies demonstrated that this is not the case [10, 30–33, 46], and on meta-analysis of four eligible studies, there was no significant difference between minimally invasive and sternotomy approaches (539 patients, OR 0.86, 95% CI 0.59–1.27, *P* = 0.45). 

Asher et al. [33] addressed this question in a cohort of 100 patients having elective primary minimally invasive AV or MV surgery compared with a matched control group undergoing conventional sternotomy. They found a similar prevalence of post-operative AF using either method, even after stratifying for valve type. However, the PAIR registry reported a 10% incidence of new-onset AF with the port access technique, which is lower than that expected for sternotomy [33].

## 8. Septic Complications

The incidence of wound infections and septic complications is lower with a thoracotomy than with a median sternotomy. Of the three studies of minithoracotomy mitral valve surgery that reported wound complications compared to median sternotomy, Grossi et al. reported an incidence of 0.9% and 5.7% for minithoracotomy and sternotomy cases, respectively (*P* = 0.05) [34]. This increased to 1.8% and 7.7%, respectively, in elderly patients (*P* = 0.03) [34], whereas Felger et al. reported no significant difference [30].

## 9. Pain, Quality of Life and Speed of Recovery

Compared with a complete sternotomy, thoracotomy incisions are associated with less pain, discomfort, and postoperative analgesics [30]. Cohn's data show less pain in hospital and after discharge, less analgesic usage, greater patient satisfaction, and a return to normal activity 4.8 weeks ahead of sternotomy patients [8].

The most insightful evidence comes from 2 studies reporting that patients undergoing surgery via a minimally invasive approach as their second procedure all thought that their recovery was faster/less painful than their original sternotomy [30, 79].

## 10. Elderly Patients

Iribarne et al. demonstrated that MIMVS can be performed safely in patients at ≥75 years old [80]. Although the minimally invasive approach was associated with slightly longer CPB and cross clamp times than was the conventional sternotomy approach, there were no significant differences in postoperative morbidity and mortality. Importantly, patients undergoing MIMVS had approximate 3 days shorter mean and 1 day shorter median durations of hospitalization, a finding that has important implications for resource use. There were significant reductions in both mean and median costs of hospitalization associated with the minimally invasive approach, a finding that correlates with the observed difference in duration of hospitalization found between the groups. In addition, patients undergoing MIMVS had faster rates for both time to independent ambulation and time to independent sit-to-stand activity [80]. Grossi et al. analyzed 111 patients undergoing MIMVS who were at least 70 years old and compared these to 259 patients having a sternotomy and concluded that this approach can be used safely in operations on the elderly population with excellent result [34]. Felger et al. recently reported 123 cases of minimal invasive mitral valve repair in patients aged ≥70 years with 1.6% operative mortality as well as 5-year actuarial survival of 87% and 5-year freedom from reoperation of 93% [30, 79]. To date, no studies have assessed any difference in postoperative functional status by type of surgery.

## 11. Hospital Stay and Costs

Some of the reported benefits of MIMVS include decreased intensive care unit a and total hospital length of stay, faster physical rehabilitation, and decreased overall hospital resource use [35, 40, 81, 82]. MIMVS is a cost-effective and cost-saving strategy for mitral valve repair and replacement compared with the traditional approach with lower cost driven largely by a decreased length of stay [80].

## 12. Conclusions

MIMVS has been proven to be a feasible alternative to the conventional full sternotomy approach with low perioperative morbidity and short-term mortality. 

Reported benefits of MIMVS include decreased postoperative pain, improved postoperative respiratory function, reduced surgical trauma, and greater patient satisfaction. Finally, compared to standard surgery, MIMVS demonstrated comparable efficacy across a range of long-term efficacy measures such as freedom from reoperation and long-term survival.

## Figures and Tables

**Table 1:** Most recent observational cohort studies of minimally invasive mitral valve surgery.

Authors	Year	Minimally invasive (number of patients)	Minithoracotomy (number of patients)	Approach	Valve	Differences
Cohn et al. [8]	1997	50	50	PS, UHS	MV, AV	Longer CBP/XT times in MI group
Navia and Cosgrove [9]	1996	31	100	MT	MV	Longer CBP/XT times, less transfusion, reduced CVA, shorter ICU/hospital stays in MI group
Glower et al. [31]	1998	21	20	MT	MV	Longer CBP/XT times, shorter return to normal activity time n MI group
Reichenspurner et al. [32]	1998	100	100	MT	MV	Reduced AF in MI group
Asher et al. [33]	1999	100	100	—	MV, AV	Longer CBP/XT times, shorter hospital stays in MI group
Grossi et al. [34]	1999	111	259	MT	MV, AV	Lower sepsis/wound complications, shorter hospital stays in MI group
Walther et al. [35]	1999	129	209	MT	MV, AV	Lower pain levels
Schneider et al. [36]	2000	21	13	MV	MT	Longer CPB time
Hamano et al. [37]	2001	21	27	PS, UHS, LHS	MV, AV	No differences
Grossi et al. [38]	2001	100	100	MT	MV	No differences
Grossi et al. [39]	2001	109	88	MT	MV, AV	Longer CBP/XT times, shorter hospital stays in MI group
Felger et al. [30]	2001	127	100	MT	MV	With AESOP shorter hospital stay time compared to ST group, shorter XC times compared to manually directed videoscope
Yamada et al. [40]	2003	66	50	LHS	MV, AV	Longer CBP/XT times in MI group
McCreath et al. [41]	2003	214	87	MT	MV	Reduced acute renal deseas in MI group
de Vaumas et al. [42]	2003	10	10	PS	MV	Longer CBP/XT times in PS group
Gaudiani et al. [43]	2004	205	616	UHS, LHS, MT	MV	Shorter hospital stay in MI repair group, less CVA in MI replacement group
Mihaljevic et al. [44]	2004	474	337	LHS, PS	MV	5-year survival better for MI group
Dogan et al. [45]	2005	20	20	MT	MV	Intraoperative complications in EABO group
Ryan et al. [46]	2005	117	117	MT	MV	Longer CBP/XT times in MI group
